# Genomically Silent Refractory Gastric Cancer in a Young Patient Exhibits Overexpression of CXCL5

**DOI:** 10.3390/curroncol29070375

**Published:** 2022-07-06

**Authors:** Jonathan Hernandez, Michael A. Turner, Prerna Bali, Mojgan Hosseini, Michael Bouvet, Kaitlyn Kelly, Marygorret Obonyo

**Affiliations:** 1 Department of Medicine, School of Medicine, University of California, San Diego, CA 92093, USA; joh013@health.ucsd.edu (J.H.); pbali@health.ucsd.edu (P.B.); 2 Department of Surgery, University of California, San Diego, CA 92093, USA; maturner@health.ucsd.edu (M.A.T.); mbouvet@health.ucsd.edu (M.B.); k6kelly@health.ucsd.edu (K.K.); 3 VA San Diego Healthcare System, San Diego, CA 92161, USA; 4 Department of Pathology, University of California San Diego, CA 92037, USA; mohosseini@health.ucsd.edu; 5 Moores Cancer Center, University of California, San Diego, CA 92093, USA

**Keywords:** young patient, advanced gastric cancer, *Helicobacter pylori*, CDH1, CXCL5

## Abstract

Gastric cancer is the third leading cause of cancer-related deaths, with more than one million new cases and approximately 841,000 deaths annually worldwide. We report a case of a young patient (25 years old) with an aggressive form of gastric cancer. The patient had previously been treated for *Helicobacter pylori* (*H. pylori*), which is a main risk factor for developing gastric cancer. Genetic testing showed an E-cadherin (*CDH1*) germline mutation of unknown significance. After eight cycles of chemotherapy, a positron emission tomography (PET) scan showed disease progression with an enlarging hypermetabolic right adnexal mass suspicious for metastatic disease. Tumor pathology demonstrated invasive and poorly differentiated gastric carcinoma. The analysis of the tumor biopsy indicated the very high expression of a chemokine, C-X-C motif chemokine 5 (CXCL5). The combination of *H. pylori* infection with an existence of a rare *CDH1* mutation could have contributed to this aggressive gastric cancer.

## 1. Introduction

Gastric cancer remains a significant global health burden as the third leading cause of cancer death and one of the most common, lethal [1], and recalcitrant malignancies [2,3]. *Helicobacter pylori* (*H. pylori)* infection is the main known risk factor for the development of gastric cancer. For most patients in the United States, gastric cancer is diagnosed in the locally advanced or late stages because screening is not performed, and the disease is only detected after the development of symptoms. Complete tumor resection, with or without adjuvant therapy, is the only curative treatment option, but only for eligible patients. As a result, most patients die within two years following operation. Most of these deaths are a consequence of gastric cancer recurrence and metastasis [3,4,5,6], with the peritoneum being the most common site of spread and treatment failure. The 5-year survival rate is less than 5% [7] after the cancer has metastasized, which has not changed significantly over the last 30 years. Despite multiple clinical trials of different treatment regimens [4,8], the prognosis remains poor for this disease. Although recent studies show that targeting the tumor microenvironment may help in developing new therapeutic treatments for gastric cancer, further studies need to be carried out to identify its complete potential [9]. In addition, even with recent advances in targeted treatments, which include trastuzumab, trastuzumab deruxtecan (T-DXd)-approved treatments for HER2-positive gastric cancer patients, and many other therapies under phase II and phase III trials, gastric cancer cure rates remain low [10,11,12,13]. Recent data from a CYTO-CHIP (Cytoreductive surgery vs. Cytoreductive surgery and Hyperthermic Intraperitoneal Therapy) study provide some evidence of treatment efficacy for aggressive gastric cancer with peritoneal metastases [14]. Here, we report a case of an aggressive and lethal gastric cancer in a young patient.

## 2. Case Report

### 2.1. Clinical Course

**Figure 1:**
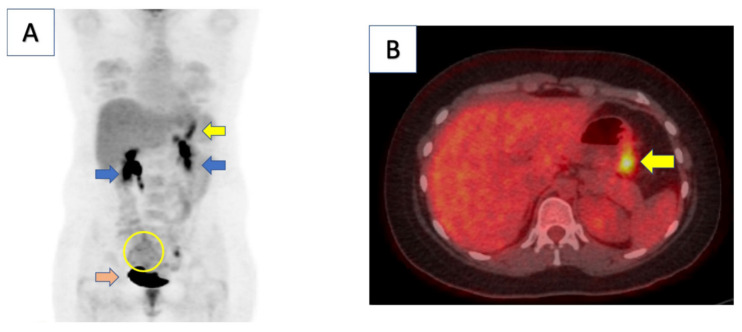
Computed tomography (CT)/positron emission tomography (PET) scan of patient demonstrated moderate focal uptake along gastric body consistent with known malignancy (yellow arrow in panel (**A**,**B**)). There was also a large, mildly hypermetabolic right adnexal area with heterogenous uptake concerning for metastatic involvement (yellow circle). Kidneys (blue arrows) and bladder (orange arrow) demonstrate physiologic uptake.

A 25-year-old female presented to the emergency department with persistent abdominal pain after finishing a standard course of treatment for *H. pylori*. Her past medical history was notable for a 2-year history of intermittent abdominal pain with bloating and a 20 lb weight loss. She was discharged with pain medication after her emergency department work up showed mild hypokalemia and a 5 mm gallbladder polyp on a right upper quadrant ultrasound. A follow-up esophagogastroduodenoscopy (EGD) 5 months later revealed a gastric ulcer. Biopsies taken at the time of the EGD were positive for gastric adenocarcinoma. Her planned gastrectomy was aborted when, upon entering the abdomen, diffuse peritoneal disease was noted, consistent with stage IV disease. Germline genetic testing showed an E-cadherin (*CDH1*) mutation at 16q22.1 (FoundationOne CDx, Foundation Medicine, Inc. Cambrige, MA, USA). The mutation was considered a variant of unknown significance (VUS). The patient received systemic chemotherapy with epirubicin, oxaliplatin, and capecitabine. After eight cycles of chemotherapy, a positron emission tomography (PET) scan showed evidence of disease progression. The patient was started on a second-line chemotherapy regimen of leucovorin calcium and irinotecan hydrochloride (FOLFIRI). A repeat PET scan 3 months later showed overall stable disease, with persistent activity in the stomach as well as an enlarging hypermetabolic right adnexal mass (Figure 1). The patient was evaluated by the surgical oncology service and presented at the multidisciplinary tumor board. Because the patient had an overall stable disease on imaging and was doing well clinically, she was determined to be a candidate for cytoreductive surgery with hyperthermic intraperitoneal chemotherapy (CRS/HIPEC). As part of her CRS/HIPEC procedure, the patient received a total gastrectomy with reconstruction and bilateral salpingo-oophorectomy as well as a distal pancreatectomy, splenectomy, and partial colectomy secondary to disease involvement. The peritoneal carcinomatosis index (PCI) was 11. The PCI is a quantitative measure of peritoneal disease burden that can range from 0 to 39, with lower numbers associated with improved survival [15].

### 2.2. Pathology

**Figure 2:**
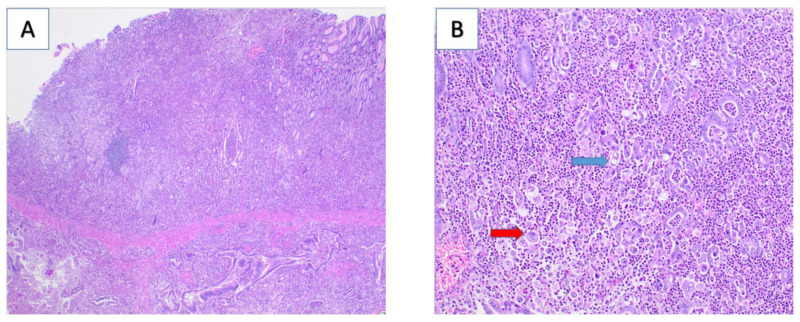
Hematoxylin and eosin staining revealed invasive, poorly differentiated gastric carcinoma invading into the muscularis propriawith minimal gland formation (panel (**A**),2× magnification). Panel (**B**) (20× magnification) demonstrates signet ring cell component (blue arrow) as well as pleomorphic neoplastic cells (red arrow).

**Figure 3:**
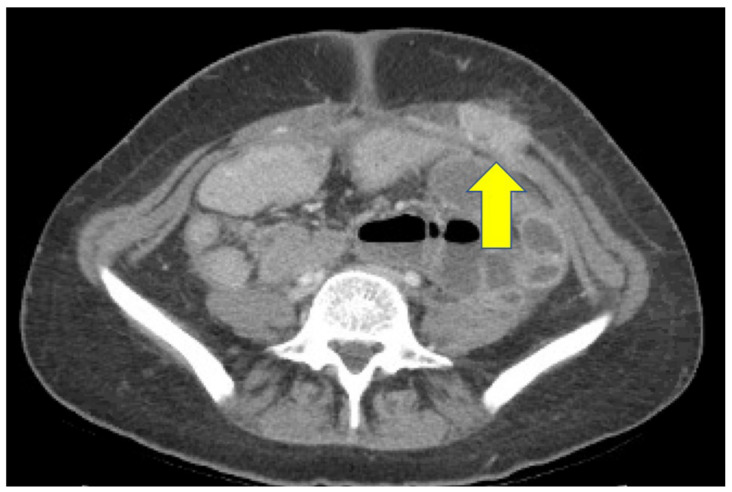
Axial CT image of case report patient demonstrated disease recurrence in left abdominal wall (yellow arrow).

Surgical pathology demonstrated invasive, poorly differentiated gastric carcinoma (Figure 2). The patient had an unremarkable recovery initially, but four months after surgery, she developed a left abdominal wall mass that was noted on interval imaging (Figure 3). During a brief hospitalization for partial small bowel obstruction, a biopsy of the mass confirmed disease recurrence. The patient received palliative radiation to her abdominal wall while hospitalized and was eventually discharged to home hospice. The patient passed away 8 months after surgery at 25 years of age.

**Figure 4:**
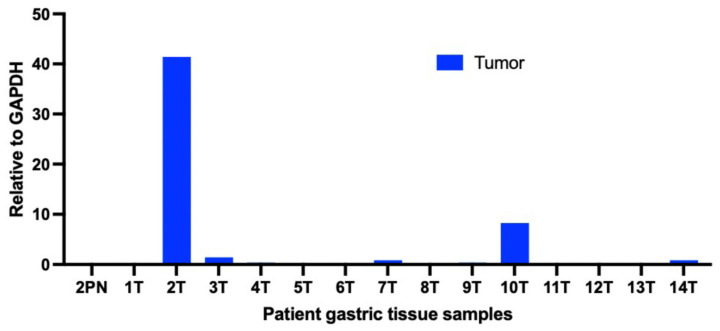
CXCL5 was overexpressed in the gastric biopsy of the case patient. CXCL5 expression in 13 other gastric biopsies is shown for comparison. CXCL5 expression was quantified via qRT-PCR and expressed relative to GAPDH using comparative cycle threshold calculations (ΔC_T_, Applied Biosystems). 2PN, paired normal control of case patient (2T); T, tumor gastric cancer tissue.

The pathology of the patient tumor showed many neutrophils in the tissue surrounding tumor cells, which led us to examine the expression of C-X-C motif chemokine 5 (CXCL5). Several studies suggest that CXCL5 is a strong neutrophil chemoattractant [16,17,18,19]. In addition, the patient had been treated for *H. pylori* infection, which is associated with the increased production of CXC chemokines [20]. The goal was to further understand the status of the disease, either indicating the extent of disease severity/malignancy or host immunity to the tumor. Total RNA was isolated from the patient’s gastric cancer tissue and processed for quantitative real-time polymerase chain reaction (qRT-PCR), as described in our previous studies [21,22,23,24], using the Direct-zol RNA mini kit (Zymo Research Corp) according to the manufacturer’s instructions. RNA quality was determined by using a Nanodrop system (Thermo Fisher Scientific, Inc., Waltham, MA, USA) followed by reverse transcription into cDNA using the High Capacity cDNA Reverse Transcription kit (Thermo Fisher Scientific, Inc.). The specific primer pairs used in the study for CXCL5 and glyceraldehyde-3-phosphate dehydrogenase (GAPDH) were as follows: forward 5′-TGGACGGTGGAAACAAGG-3′; reverse, 5′-CTTCCCTGGGTTCAGAGAC-3′ [25] and forward, 5′-CCTGGTCACCAGGGCTGC-3′; reverse, 5′-CCGTTCTCAGCCTTGACGG-3′ (Integrated DNA Technologies, Inc., Clareville, IA, USA), respectively. The expression of CXCL5 for each sample was expressed relative to its GAPDH using comparative cycle threshold calculations (ΔC_T_, Applied Biosystems, Waltham, MA, USA) and plotted using GraphPad Prism software. Including this case, we examined the expression of CXCL5 in a series of 13 other gastric tissue samples obtained from the UCSD Cancer Center Biorepository and new presenting patients at our center for comparison. All patients provided written informed consent and were followed up. The additional gastric tissue samples for comparison included 13 gastric cancer tumor tissues (T). A summary of all patients and their gastric tumor characteristics, including the case patient (2T), are provided in Table 1. There was very high expression of CXCL5 in the tumor of the case patient (Figure 4). Among the gastric tissue samples we analyzed, only one other sample from an older patient (10T) had significant CXCL5 levels (over 2-fold relative to GAPDH). However, the levels were much lower than the levels observed in the patient described in this case report.

## 3. Discussion

This is a very rare case of early-onset, aggressive, poorly differentiated gastric carcinoma without a well-characterized pathogenic germline alteration. Gastric cancer is a disease that primarily affects older adults with a median age of 68 years at diagnosis in the United States [26,27]. The diagnosis of gastric cancer is often delayed as patients present with non-specific abdominal complaints. Upper GI endoscopy is the preferred method for the evaluation of a suspicious gastric lesion as it allows for tissue diagnosis [28]. Endoscopic ultrasound (EUS) was thought to be more sensitive for staging gastric cancer in the T and N stage; however, with advances in computed tomography (CT) imaging techniques, CT imaging is just as accurate [29,30,31,32]. As such, CT imaging is now the most common imaging technique for the staging of gastric cancer as it can assess tumor invasion, lymph node involvement, and the presence of distant metastasis [28]. PET/CT is also useful for assessing metastatic disease or recurrence [28]. However, a high number of patients are still found to have metastatic disease upon staging laparoscopy, which was unappreciated in cross sectional imaging [33]. One series from Memorial Sloan Kettering found that as many as 37% of patients thought to have localized gastric cancer with CT or endoscopic ultrasound, had metastatic disease discovered with a staging laparoscopy [34].

There are two classification systems for the histology of gastric cancer: Lauren’s criteria and the World Health Organization (WHO) system. Lauren’s criteria divides gastric cancer into two types: intestinal and diffuse type [35]. The intestinal type is more often associated with environmental risk factors and more often affects older males [35]. The diffuse type is more often associated with genetic risk factors and more often affects younger patients and females [35]. Of the two, the diffuse type has a worse prognosis. The WHO classification system identifies four major types of gastric cancer histology: tubular, papillary, mucinous, and poorly cohesive, with tubular pathology being the most common [35]. The WHO classification also recognizes several less common histological types: adenosquamous carcinoma, squamous carcinoma, hepatoid adenocarcinoma, carcinoma with lymphoid stroma, choriocarcinoma, parietal cell carcinoma, malignant rhabdoid tumor, mucoepidermoid carcinoma, Paneth cell carcinoma, undifferentiated carcinoma, mixed adeno-neuroendocrine carcinoma, endodermal sinus tumor, embryonal carcinoma, pure gastric yolk sac tumor, and oncocytic adenocarcinoma [35]. The case patient’s histologic type was described as a poorly differentiated/diffuse carcinoma.

We showed the overexpression of CXCL5 in the gastric tumor of the case patient. There is some recent evidence from clinical studies indicating that chemokines may play an important role in the development and progression of gastric cancer [36,37]. Certain chemokines, therefore, may potentially function as future biomarkers to stratify treatment for patients. The expression of CXCL5 has been implicated in the pathogenesis and progression of several solid tumors, including colorectal cancer [38,39], breast cancer [40], hepatocellular carcinoma [19], bladder cancer [41], pancreatic cancer [42], lung cancer [43], prostate cancer [44], and gastric cancer [18,45,46,47,48]; however, these were in older patients. Among the 14 gastric tumor tissue samples we analyzed, the expression of CXCL5 was greatest in the gastric tissue biopsy from the case patient. To our knowledge, this is the first report of high CXCL5 expression in a young gastric cancer patient. Given that this was a young patient with a histologically diffuse tumor type, the implication is likely that this could be related to genetic predisposition. It is possible that the current *CDH1* VUS could turn out to be of particular importance in aggressive gastric cancer. The accumulation of similar cases in the future will allow for further analysis of this observation. In addition, given that the patient had been treated for *H. pylori*, it is possible that the combination of *H. pylori* infection and the presence of this yet unproven, but possibly putative pathogenic *CDH1* germline alteration contributed to the fast and aggressive form of gastric cancer in this patient.

In conclusion, this was a rare case of a young patient with a germline *CDH1* VUS with advanced gastric cancer that proved to be refractory to existing therapies, including systemic and intraperitoneal chemotherapy and complete cytoreduction. The tumor exhibited the very high expression of CXCL5. This is a hypothesis-generating association, and further investigations to determine if there is a link between CDH1 alterations and CXCL5 overexpression are warranted. Such an association may provide further insight into genomically silent, treatment refractory, and poorly differentiated gastric cancer.

## Figures and Tables

**Table 1:** Characteristics of gastric cancer patients.

Patient ID	Patient Sex	Patient Age	Patient Race/ Ethnicity	Primary	Grade	Metastatic	Stage	Chemotherapy
2PN	F	25	Hispanic	Adenocarcinoma, diffuse type	G3: poorly diff	Yes	IV (ypT4bypN3bypM1)	EOX/FOLFIRI
1T	F	53	Asian	Adenocarcinoma, signet ring-cell	G3: poorly diff	No	IIA (ypT3ypN0)	EOX
2T	F	25	Hispanic	Adenocarcinoma, diffuse type	G3: poorly diff	Yes	IV (ypT4bypN3bypM1)	EOX/FOLFIRI
3T	M	66	Asian	Adenocarcinoma, residual	G3: poorly diff	No	IIA (ypT3N0)	EOX and chemorads with capecitabine
4T	M	51	White	Adenocarcinoma	G3: poorly diff	No	IIB (ypT4aN0)	Yes (unspecified in notes)
5T	M	78	White	Invasive adenocarcinoma	G3: poorly diff	Yes	IIIC (pT4aN3a)	No
6T	F	49	White	Invasive adenocarcinoma, signet ring	G3: poorly diff	Yes	IIB (pT4aN0)	No
7T	M	69	Hispanic	Adenocarcinoma	G3: poorly diff	Yes	IV (ypT4bN3bM1)	FOLFOX
8T	F	48	Asian	Adenocarcinoma, diffuse type. Signet-ring	G3: poorly diff	No	IIIC (pT4aN3a)	No
9T	F	81	Vietnamese	Gastric adenocarcinoma, intestinal type	Moderate to poorly differentiated	Invades serosa	pT4aN0	No
10T	M	81	Asian	Gastric adenocarcinoma	G3: poorly differentiated	Yes	ypT3N3a	FOLFOX
11T	F	83	Hispanic	Gastric adenocarcinoma	G3: poorly differentiated	No	mpT2N3a	No
12T	M	73	White	Gastric adenocarcinoma	G3: poorly differentiated, undifferentiated	No	ypT3N1	FLOT/FOLFOX (neoadjuv)
13T	F	66	Asian	Gastric adenocarcinoma, diffuse type with signet ring	G3: poorly differentiated, undifferentiated	No	ypT4aN0	FLOT
14T	F	66	White	Gastric adenocarcinoma with signet ring cell	G3: poorly differentiated	Yes	pT4aN3b	No

2PN = paired normal control of case patient (2T), T = tumor gastric cancer tissue, EOX = epirubicin, oxaliplatin, capecitabine, FOLFIRI = folinic acid, fluorouracil, and irinotecan, FOLFOX = folinic acid, fluorouracil, and irinotecan, FLOT = fluorouracil, leucovorin, oxaliplatin, and docetaxel.

## Data Availability

The data were presented at the Digestive Disease Week Conference, 21–24 May 2022, San Diego, CA, USA.
